# Molecular modeling and simulation of three important components of Plant Pathogen Interaction cascade in Vigna mungo

**DOI:** 10.6026/97320630013323

**Published:** 2017-10-31

**Authors:** Pankaj Kumar Singh, Anju Patel, Sayak Ganguli, Amita Pal

**Affiliations:** 1 Division of Plant Biology, Bose Institute, Kolkata-700054; 2 AIIST, Palta & The Biome, Kolkata- 700064

**Keywords:** MYMIV, MAP kinase, WRKY 33, Simulation, Transcriptome

## Abstract

Plant pathogen interaction plays a great role in plant immunity. The regulation of various components of plant pathogen interactions is quite complicated and is very important in establishing relationship among components of this system. Yellow Mosaic Disease is common among legumes such as Vigna mungo. Mungbean Yellow Mosaic India Virus (MYMIV) and whitefly (Bemisia tabaci) is a vector causing the disease. Therefore, it is of interest to document the molecule models of three different components of Plant Pathogen interaction cascade- MAP kinase1, MAP kinase 2 and WRKY33 from V. mungo resistant to MYMIV. Both the MAP kinases were sequenced for this study while WRKY 33 was extracted and modeled from transcripts generated from two different transcriptome libraries, one set MYMIV- challenged, the other fed with aviruliferous whitefly. Post simulation studies revealed that MAPKs contained less percentage of disordered residues and were structurally more stable and than WRKY33.

## Background

Legumes are economically important crops consumed for food and forage owing to high protein content. Legumes also have the nitrogen fixation ability that augments soil fertility. Among various legumes, Vigna mungo (black gram) constitutes a significant portion of diet in South East Asian countries. Though, India is the largest producer of V. mungo, the average yield of this crop is low mainly due to various biotic and abiotic stresses [1]. Among these Yellow Mosaic Disease (YMD) caused by Mungbean Yellow Mosaic India Virus (MYMIV) is a major menace responsible for huge penalty in crop yield of several grain legumes including V. mungo. In the current scenario, knowledge of molecular mechanism of immune responses of V. mungo is the primary focus towards the crop improvement programme. In our previous study we have identified differentially expressed ESTs that were modulated under MYMIV stress [2]. Significant number of such ESTs falls under the category of signal transduction. One of the keys signaling components identified in the suppression subtractive hybridization (SSH) library was MAP kinase [3]. Expression of one of these MAPKs was up regulated in the resistant genotype of V. mungo and down regulated in the susceptible genotype upon MYMIV inoculation and designated as VmMAPK1 [3]. In plants, MAPKs regulate vital physiological functions including cell growth and differentiation, hormonal regulation and development [4]. These are also involved in transducing stress signals to downstream molecules through a cascade of phosphorylation and dephosphorylation of a wide range of substrates [5]. Although MAP kinases in crops like rice, tomato, maize, cotton are well explored, information in leguminous crops is limited. Moreover, evidences of MAP kinases related to virus immunity are inadequate.

On the contrary, extensive studies have illustrated the function of stress related MAPKs in model plants. The role of Arabidopsis MPK3 and MPK6, downstream of the receptor FLS2 against bacteria and fungi is well demonstrated in the protoplast system [6]. MAPK-mediated phosphorylation of WRKY8 has been shown to play an important role in the defense response in tobacco. About 17 members in rice MAPK and 20 MAPKs in Arabidopsis have been identified, however only two MAPKs are identified and one of them characterized in V. mungo [3]. Recently we have shown that VmMAPK1 is a potential signaling player though functional characterization of the gene to gain an insight into Vigna-MYMIV interaction [3]. In addition the indication obtained from our earlier study on modulation of transcripts during incompatible interaction between the host and the virus [2] we anticipated that expression of VmMAPK1 plays a crucial signaling role in triggering immune response by inducing downstream pathogenesis related genes via activation of transcription factors. On the above backdrop, this study focuses on molecular modeling and simulation of VmMAPK1, VmMAPK2 and WRKY 33. Two different WRKY DNA binding proteins were predicted from transcriptome library prepared from a resistant variety of V. mungo. Two different libraries were for MYMIV challenged (infected with viruliferous whitefly Bemisia tabaci) and another set being mock inoculated (fed with aviruliferous whitefly).

## Methodology

### MAPkinase

**Figure 1:**
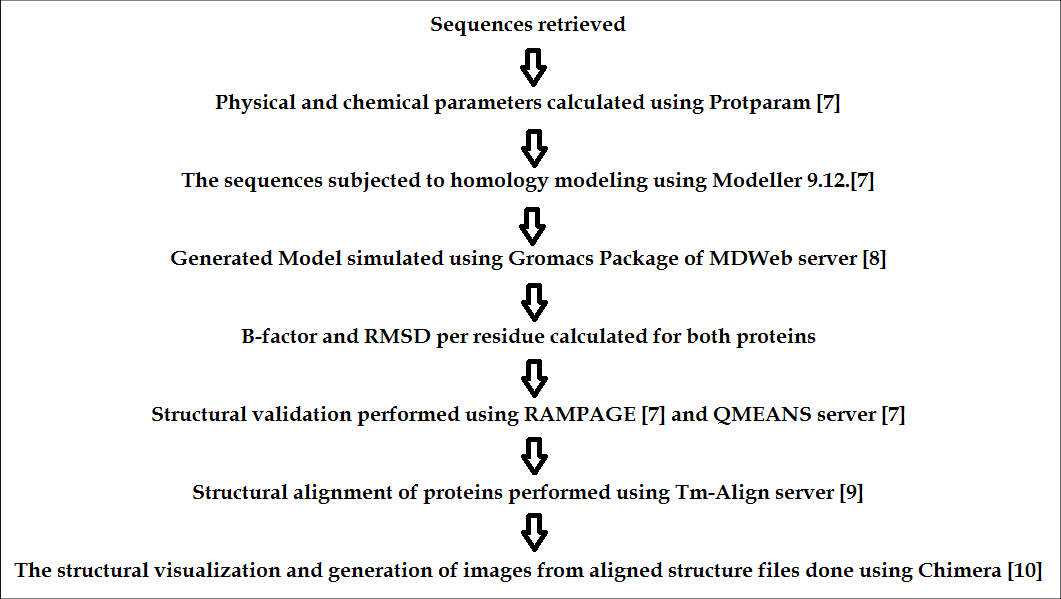
General Computational Workflow

Data collection, isolation and sequencing of MAPkinase Figure 1 followed by submission to Genbank were done according to the protocol described in Patel et al. [3]. Accession numbers AID46462.1 (VmMAPK1) and AID46463.1 (VmMAPK2).

### WRKY33

RNA isolation and sequencing of samples for both mock control (MC) and MYMIV- inoculated (MI) were done according to the protocol described in Ganguli et al. [11]. The library SRX1032950 and SRX1082731 were submitted in Sequence Read Archive (SRA) for the MC and MI reads, respectively.

### De-novo assembly and transcript generation

Quality controlled Illumina HiSeq 2000 reads were de-novo assembled using Velvet1.2.10 tool [12] to get contigs. Next, the assembled contigs were processed through Oases 0.2.08 [13] pipeline to generate transcripts. After that, combined transcripts were clustered (at 95% identity) using CD-HIT tool [14] to generate unigenes. Moreover, differential gene expression analysis was done using DeSeq tool to decipher the mode of unigene expression in mock control (MC) and MYMIV inoculated (MI) samples.

### Clusters of Orthologous Groups (COG) and GO categorization

The COG database was downloaded from https://www.ncbi.nlm.nih.gov/COG/ and the unigene sequences were compared using standalone BLASTX [15] with an E-value cutoff of 1.0E - 5, and best 26 categories were computed. Functional annotation of the transcripts was performed using BLAST2GO tool to categorize the transcripts into respective gene ontologies using controlled vocabularies [16] in order to recover GO terms with their BLASTx description. Pathway mapping of the annotated transcripts was done using the KAAS server against the KEGG database (Kyoto Encyclopedia of Genes and Genomes) (http://www.genome.jp/kegg) to identify the respective pathways. Sequences of the WRKY33 transcripts were also submitted to ORF Finder and longest ORF was selected for further analyses.

## Result and Discussion

**Figure 2:**
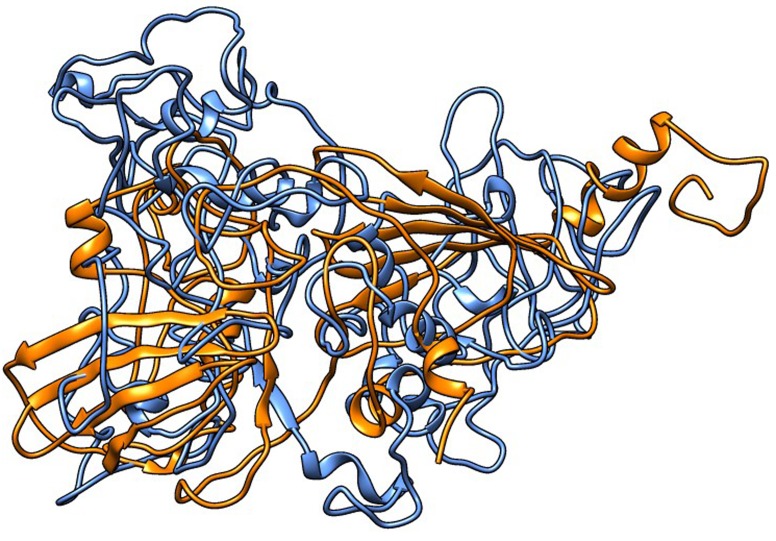
Alignment of WRKY 33(MC)- Orange and WRKY 33(MI)- Blue

A large number of residues in WRKY33 were observed to be unstructured in comparison to MAP kinases. A huge improvement in structure was noted between the structures of proteins when both pre and post simulation structures were compared Figure 2. Again changes were more prominent in case of WRKY33 than VmMAP kinases.

The structural alignment for MAPkinases revealed a RMSD score of 1.63 and identical: aligned ratio was found to be 0.676, on the other hand the RMSD value for WRKY33 alignment was computed to be 6.53 and identical: aligned ratio was 0.056. Low value of RMSD for MAPkinases highlights the structural similarities and conservation in the structure of the proteins, while high value of RMSD reflects the variation in structure of WRKY33 proteins of both MC and MI. These results are also supported by similar values for identical: aligned residue ratio.

The results of structural and sequence based analysis of protein highlights different structural and functional features of proteins. Of all four proteins MPK1 posses most stable structure, which is reflected by its low unstructured residue percentage, low instability index, high aliphatic index and better Gravy (grand average of hydropathicity) value. While on the other side WRKY33 MI structure seems to be very unstable which can be interpreted from high unstructured residue percentage and high instability index, low Aliphatic index and GRAVY value Table 1. The hierarchy of structural stability can be represented as: VmMPK1>VmMPK2>WRKY 33 MC> WRKY33MI.

**Figure 3:**
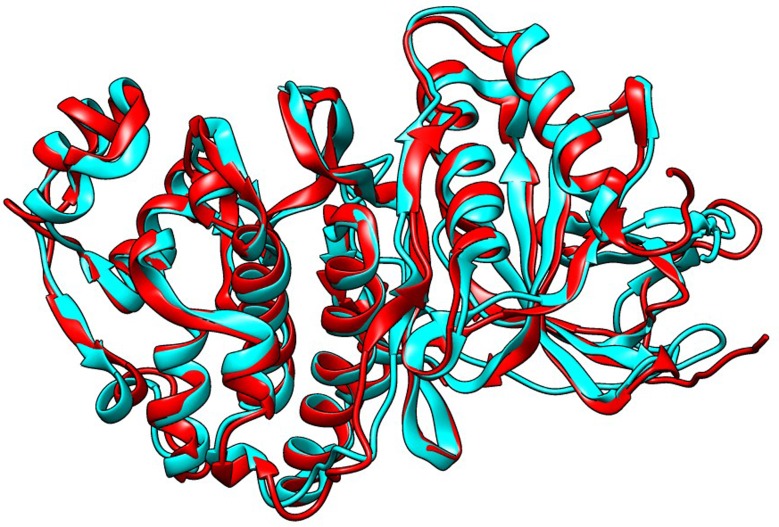
Alignment of VmMAP kinase 1-Cyan and VmMAP kinase 2-Red.

MAP kinases have been studied in great detail using crystallography and it has been observed that they have significant conserved structural features Figure 3. Functionally they are much less dynamic as compared to the WRKY33 proteins since they unequivocally target specific residues - serine/threonine in the dipeptide motif S/T-P that is often regulated by the presence or absence of leucine at -1 or -2 or - 3 positions [17]. Their interactions with scaffolding proteins and other interactors of the signalling cascades are specific and hence the structural variations are not required to accommodate multiple random partners [18].

**Figure 4:**
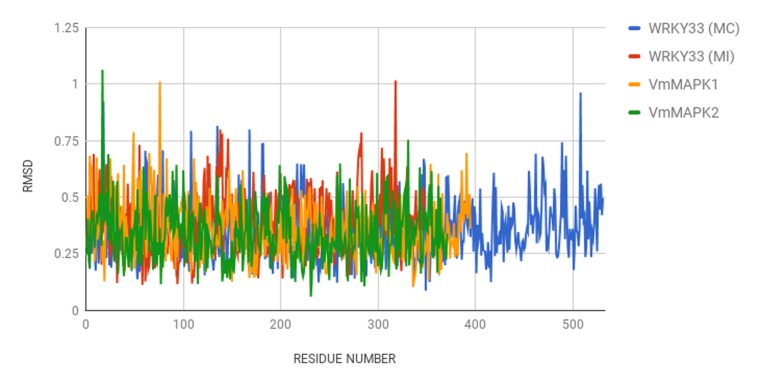
RMSD value per residue of all four proteins.

**Figure 5:**
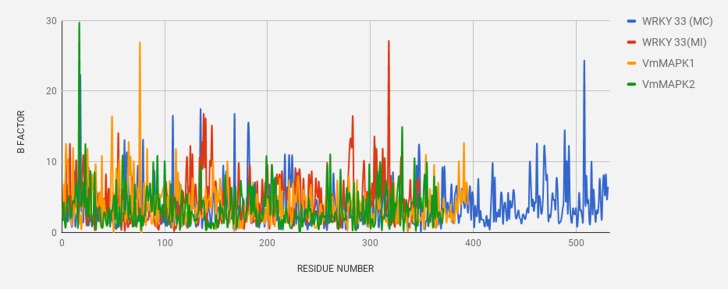
B Factor per residue of all four proteins

The variation in structural dynamics Figure 4 and 5 of the WRKY proteins from both the samples probably is an indicator to the numerous functions that they perform in the cell under different biotic and abiotic stress responses or during their role in secondary metabolism. Apart from this they are also involved in various crosstalk, which regulate response to stress and development [19]. Numerous reports suggest that they are also regulated by a plethora of plant enzymes in various elucidated and unelucidated mechanisms [20]. The variability of the interactors both upstream and downstream of a particular cascade probably requires the structure of these factors to be dynamic to accommodate the differential binding affinities of its interacting partners.

## Conclusion

Our analyses revealed interesting features both in sequence composition leading to structural specifications. MAP kinase structures predicted and simulated in the study conform to the established structures of MAP kinases revealing the standard features. WRKY33 proteins from the two samples display a greater variation and dynamic structure brought about by large regions of unstructured residues. These structures should enable further studies in molecular interactions with different substrates of the proteins under various stress conditions.

## Figures and Tables

**Table 1:** Tabular representation of results of structural and sequence based analysis of all four proteins

S. No	Protein Name	Unstructured Residues (%)	Average RMSD	Average B-Factor	Instability Index	Aliphatic Index	GRAVY
1	VmMAPK1	13.13	0.361	3.855	42.35	88.96	-0.295
2	VmMAPK2	6.99	0.339	3.478	48.41	89.89	-0.352
3	WRKY33 MI	66.85	0.392	4.579	64.29	42.63	-0.894
4	WRKY33 MC	72.79	0.356	3.725	60.55	44.09	-0.783
